# Associations of changes in waist-to-height ratio with all-cause mortality among Chinese older adults: a national cohort study

**DOI:** 10.3389/fnut.2026.1839686

**Published:** 2026-05-14

**Authors:** Juanxia Miao, Xue Wang, Kun Huang, Yiwen Wang, Shuang Zang

**Affiliations:** 1 Shanxi Provincial People’s Hospital, Taiyuan, Shanxi, China; 2 Department of Community Nursing, School of Nursing, China Medical University, Shenyang, Liaoning, China; 3 School of Nursing, Peking University, Beijing, China; 4 Department of Ultrasound, The First Hospital of China Medical University, Shenyang, Liaoning, China

**Keywords:** all-cause mortality, body mass index, Chinese, older adults, waist-to-height ratio

## Abstract

**Background:**

Waist-to-height ratio, as an anthropometric indicator, is considered a valid tool for assessing the health status of older adults. However, little is known about the associations between changes in waist-to-height ratio and all-cause mortality in older adults. The aim of the study was to investigate the association between changes in waist-to-height ratio and all-cause mortality in Chinese older adults.

**Method:**

This study was based on the Chinese Longitudinal Healthy Longevity Survey from 2011 to 2018, including a sample of 4,065 participants aged 60 years and over. Changes in waist-to-height ratio were divided into quintiles, with waist-to-height ratio-stable group (change less than 0.01) defined as the reference group. Cox proportional hazards models were used to examine the associations between changes in waist-to-height ratio and all-cause mortality, adjusted for covariates including demographic characteristics, health behaviors, and health status. Kaplan–Meier survival curves were used to study the associations between changes in different categories of waist-to-height ratio and all-cause mortality.

**Results:**

The study documented 1,449 deaths among the 4,065 participants. Changes in waist-to-height ratio were significantly associated with all-cause mortality (adjusted hazard ratio: 0.30, 95% confidence interval: 0.13, 0.68). Compared with participants within waist-to-height ratio-stable group, a decline in waist-to-height ratio of ≥ 0.06 was associated with increased all-cause mortality (adjusted hazard ratio: 1.45; 95% confidence interval: 1.24, 1.71). The Log-rank statistic showed the largest decrease in waist-to-height ratio and the lowest cumulative survival. There was no significant interaction between the study subgroups.

**Conclusion:**

There was an inverse association between changes in waist-to-height and all-cause mortality. Compared with waist-to-height ratio-stable group, participants who had a decline more than 0.06 in waist-to-height ratio had an increased risk of all-cause mortality. This suggested that changes in waist-to-height ratio may be a simple and effective indicator of mortality risk for older adults.

## 1 Introduction

With the aging of the population, the health of older adults is a matter of increasing concern. The causes of death among older adults are mainly chronic diseases (1). To prevent and control these chronic diseases, effective risk factors and assessment indicators need to be found. There was growing evidence that anthropometric indicators [e.g., weight, waist circumference, body mass index (BMI)] were associated with all-cause mortality (2). Recent studies have shown that waist-to-height ratio (WHtR) is considered a more sensitive and accurate indicator of obesity compared with weight, waist circumference, and BMI (3–5). WHtR is a simple and practical assessment of obesity and metabolic syndrome that reflects the distribution and metabolic function of abdominal fat (6–8). WHtR is the ratio of waist circumference to height, which accounts for differences in height and is not affected by ethnicity, age, or gender (9). WHtR may be a clinically useful indicator of nutritional status and muscle mass in older adults, which is due to its quick, easy, and inexpensive nature, and only a tape is needed for measurement.

Currently, there is controversy regarding the association between WHtR and mortality risk. A study conducted on the Japanese population elucidated a U-shaped association between WHtR and all-cause mortality in male, with no observed association in female (10). In the German population, lower or higher WHtR was associated with increased all-cause mortality in male, whereas in female, only higher WHtR showed a significant association with all-cause mortality (11). Another study has shown a positive association between higher WHtR levels and increased risks of cardiovascular diseases and all-cause mortality (12). Contrarily, a study focusing on participants aged 80 and above showed an inverse association between elevated abdominal fat accumulation (as assessed by waist circumference and WHtR) and cardiovascular and all-cause mortality (13). It is noteworthy that as participants age, a gradual and progressive decline in weight is observed (14). And patients with wasting chronic diseases, such as malignant tumors and lung diseases, were often observed to have weight loss as the disease progressed. Changes in anthropometric indicators may provide a more comprehensive assessment of the effects of excess fat (15). Most of the available studies have focused on the association between changes in weight, waist circumference, BMI and all-cause mortality (16). However, there is currently a scarcity of studies on the association of changes in WHtR with all-cause mortality, especially in Chinese older adults.

Therefore, this study aimed to explore the associations between changes in WHtR and all-cause mortality among older adults in China using data from the Chinese Longitudinal Healthy Longevity Survey (CLHLS). This provides evidence and recommendations for health management and prevention strategies for older adults.

## 2 Methods

### 2.1 Study participants

The CLHLS is an ongoing, prospective, nationally representative longitudinal survey of community-dwelling older adults in China that began in 1998, with the follow-up surveys conducted every 2–3 years. The CLHLS employed a targeted, multi-stage cluster random sampling design to ensure the representativeness of the sample and a balanced distribution of age and gender. Additionally, using the random selection method, half of the counties and cities were selected from 23 of the 31 provinces, autonomous regions, and municipalities in mainland China, covering approximately 85% of the Chinese population in terms of geographic distribution (17). All data were obtained from face-to-face structured questionnaire interviews and physical examinations conducted by a trained survey team in participants’ homes (18, 19). Detailed descriptions of the sampling design and data quality control procedures of the CLHLS have been reported in previous studies by Zeng et al. (18, 20).

**Figure 1:**
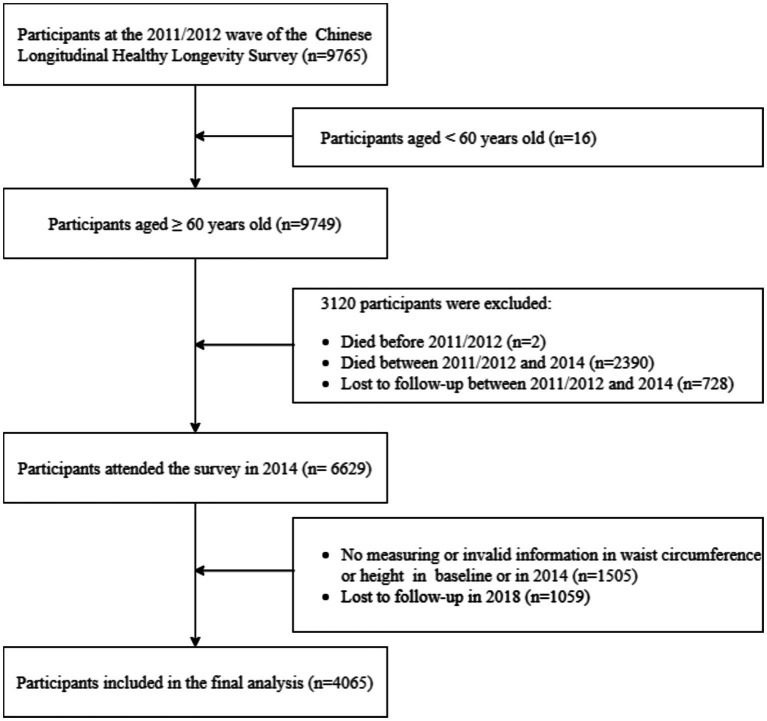
Derivation of the study participants from the Chinese Longitudinal Healthy Longevity Survey.

Considering that the measurement of waist circumference was added to the questionnaire from 2011, the study selected data from the 2011–2018 CLHLS waves. A total of 9,765 participants were initially included at baseline (2011/2012 survey). The final analytical sample was derived by applying predefined eligibility criteria and restricting the analysis to participants with complete anthropometric and follow-up data. First, according to the World Health Organization’s age classification criteria, the starting age for older adults in developing countries, including China, is 60 years old (21); thus a total of 16 participants below the age of 60 were excluded from this study. Then we excluded 2 participants who died before the 2011/2012 survey and 3,120 participants who died or were lost to follow-up between the 2011/2012, and 2014 surveys. A total of 1,505 participants were excluded due to null values or logical errors in waist circumference, height, and weight in their baseline data or in the 2014 survey data. We further excluded 1,059 participants who were lost to follow-up in the 2018 survey. At last, a total of 4,065 participants were included in this study (Figure 1).

### 2.2 Ethics approval and consent to participate

The CLHLS study obtained ethical approval from the Biomedical Ethical Committee of Peking University (IRB00001052-13074). All participants signed written informed consent forms before participating in the survey and voluntarily agreed to participate. The Declaration of Helsinki guidelines and regulations were followed in all aspects of the study.

### 2.3 Anthropometric measures

The well-trained survey team measured the waist circumference [centimeters, (cm)] and height (cm) of participants during the investigation. When measuring the waist circumference, participants were asked to stand up straight and relax the abdomen with gentle breathing, and the measurement was taken at the end of the exhalation. The midpoint between the lower rib margin and the iliac line was selected as the measurement site. A soft non-stretchable tape measure was used to take two measurements and the average value was taken (22). WHtR was calculated by dividing the waist circumference of a person by his/her height. The changes in WHtR were calculated by subtracting the values obtained in the 2011/2012 survey from the values obtained in the 2014 survey. The changes in WHtR were categorized into five groups (≤−0.06, −0.05 to −0.02, −0.01 to 0.01, 0.02 to 0.05, and ≥ 0.06), representing substantial decrease, moderate decrease, stable change, moderate increase, and substantial increase, respectively. This classification was based on a quantile-oriented approach informed by previous studies (23, 24) and the percentile distribution of WHtR changes in the study population. This approach ensures adequate sample size within each category for stable statistical estimation.

### 2.4 Outcome

In this study, the outcome variable was all-cause mortality. The participants’ information on vital status (survival time and survival status) was ascertained through the follow-up surveys. The survival time was quantified in months and calculated from the date of the CLHLS interview in 2014 to either the date of death or the last interview in 2018. The documentation of death was mostly sourced from official death certification, but sometimes from next-of-kin or other informants, such as the village doctor of the deceased participant (17, 25).

### 2.5 Covariates

Based on previous studies, this study controlled for a series of sociodemographic characteristics, health behaviors, and health status covariates (22, 26, 27), including baseline WHtR, age, sex (male or female), urban–rural distribution (rural or urban), race (Han or other), living arrangement (with household members, living alone, or pension institutions), education level, marriage status (have a spouse or no spouse), self-reported quality of life (good or bad), self-reported health (good or bad), smoking status (no or yes), alcohol consumption (no or yes), regular exercise (no or yes), hypertension (no or yes), diabetes (no or yes), heart disease (no or yes), stroke or cerebrovascular (no or yes), cancer (no or yes), and dyslipidemia (no or yes).

### 2.6 Statistical analysis

The characteristics of the participants were compared using analysis of variance test for continuous variables and the Chi-square test for categorical variables, according to the categories of changes in WHtR. Based on the visual examination of Q-Q plots, continuous variables that conformed to normal distribution were expressed as the mean ± standard deviation (SD), while the other continuous variables that did not conform to normal distribution were expressed as the median (P25, P75). For categorical variables, they were presented as numbers (percentages).

The Cox proportional hazards models were utilized to examine the association between changes in WHtR and all-cause mortality. The changes in WHtR and categories of the changes in WHtR were included as independent variables, and the participants with the changes in WHtR stable (−0.01–0.01) were the reference group. The results were presented as hazard ratios (HRs) with 95% confidence intervals (95% CIs). Model 1 was not adjusted for any variables. Model 2 was adjusted for baseline WHtR. Model 3 was further adjusted for socioeconomic variables: age, sex, urban–rural distribution, race, living arrangement, education level, and marriage status. Based on model 3, model 4 was further adjusted for health behaviors and health status: self-reported quality of life, self-reported health, smoking status, alcohol consumption, regular exercise, hypertension, diabetes, heart disease, stroke or cerebrovascular, cancer, and dyslipidemia.

Kaplan–Meier survival curves were used to investigate the associations of categories of the changes in WHtR with all-cause mortality. The Log-Rank statistic was used to analyze the significance of these associations between categories of the changes in WHtR.

Stratified and interaction analyses were performed to further examine whether the association between the changes in WHtR and all-cause mortality differed by sex, race, self-reported quality of life, self-reported health, smoking status, alcohol consumption, regular exercise, hypertension, diabetes, heart disease, stroke or cerebrovascular, cancer, and dyslipidemia.

Additionally, a series of sensitivity analyses were performed. Firstly, due to missing data at baseline and follow-up, multivariate imputations by chained equations and Cox proportional hazards models were performed. Secondly, to prevent potential bias of data due to early death, participants who died within 6 months of the follow-up were excluded (28). Thirdly, to limit potential confounding of pre-existing wasting diseases and lipid metabolic disease, participants who suffered from diabetes or dyslipidemia were excluded (22, 29). Finally, we explored the association of BMI changes and the categories of BMI changes with all-cause mortality.

In the sensitivity analyses, BMI changes were used in this study as a variable and expressed as a percentage, which was calculated as changes in BMI between the baseline and follow-up BMI divided by the baseline BMI. Then the study categorized the percentage of BMI changes into five groups: <−10, −10% ~ −5, −5% ~ +5%, +5% ~ +10%, and > + 10%.

The statistical analyses were conducted using SPSS 25.0 (IBM SPSS Inc., Chicago, United States), Empower (R) (www.empowerstats.com, X&Y solutions, Inc. Boston MA), and R.^1^ Two-tailed *p* values less than 0.05 were considered statistically significant.

## 3 Results

### 3.1 Baseline characteristics of study participants

A total of 4,065 participants were finally included in the study and were divided into five groups according to the changes in WHtR. The changes in WHtR of 778 participants (19.14%) remained within 0.01 during the follow-up, 777 participants (19.11%) had a 0.02 to 0.05 loss in WHtR, and 907 participants (22.31%) lost more than 0.06. Conversely, 819 participants (20.15%) had a 0.02 to 0.05 gain in WHtR, and 784 participants (19.29%) gained more than 0.06. Among these participants, 1,449 (35.65%) mortality events were recorded. All participants’ mean age was 81.43 years (SD: 10.26) at baseline, and the females accounted for 51.69%. Compared with those who remained within 0.01 in the changes in WHtR, those with WHtR loss greater than 0.06 were older, predominantly female, more likely to have no spouse, and lived in urban. However, the participants’ the changes in WHtR who remained within 0.01 were more likely to be non-smokers and non-drinkers. On the other hand, they were more likely to report poor quality of life and health status (Table 1).

**Table 1:** Baseline characteristics of the study participants by the categories of changes in waist-to-height ratio.

Variables	Changes in Waist-to-Height Ratio						*P*
	All	≤−0.06	−0.05 ~ −0.02	−0.01 ~ 0.01	0.02 ~ 0.05	≥0.06	
*N* (%)	4,065 (100.00)	907 (22.31)	777 (19.11)	778 (19.14)	819 (20.15)	784 (19.29)	
Age, (years), mean (SD)	81.43 ± 10.26	82.94 ± 10.06	80.66 ± 10.29	80.19 ± 9.68	80.82 ± 10.16	82.32 ± 10.21	<0.001
Survival time, (months) Median (P25, P75)	109.75(82.58, 129.00)	114.50(70.42, 129.79)	109.75(93.50, 128.83)	108.17(97.27, 129.00)	109.58(90.38, 128.29)	109.75(80.27, 128.58)	0.945
Waist-to-height ratio, Mean (SD)	0.53 ± 0.08	0.58 ± 0.07	0.54 ± 0.06	0.52 ± 0.06	0.51 ± 0.06	0.47 ± 0.08	<0.001
Education level, (years), Median (P25, P75)	0 (0, 5.00)	0 (0, 4.00)	0 (0, 5.00)	1.00 (0, 5.00)	0 (0, 4.00)	0 (0, 4.00)	<0.001
Sex, (*n*, %)							<0.001
Male	1964 (48.32)	362 (39.91)	399 (51.35)	427 (54.88)	434 (52.99)	342 (43.62)	
Female	2,101 (51.69)	545 (60.09)	378 (48.65)	351 (45.11)	385 (47.01)	442 (56.38)	
Race, (*n*, %)							0.050
Han	3,383 (93.43)	772 (91.67)	645 (94.16)	630 (94.74)	668 (92.78)	668 (94.48)	
Other	238 (6.57)	72 (8.53)	40 (5.84)	35 (5.26)	52 (7.22)	39 (5.52)	
Urban–rural distribution, (*n*, %)							0.020
Rural	2,336 (57.47)	505 (55.68)	450 (57.92)	439 (56.43)	511 (62.39)	431 (54.97)	
Urban	1729 (42.53)	402 (44.32)	327 (42.09)	339 (43.57)	308 (37.61)	353 (45.03)	
Living arrangement, (*n*, %)							0.371
With household members	3,224 (80.26)	713 (79.13)	607 (79.24)	629 (82.22)	665 (82.00)	610 (78.81)	
Living alone	745 (18.55)	174 (19.31)	151 (19.71)	130 (16.99)	139 (17.14)	151 (19.51)	
Pension institutions	48 (1.20)	14 (1.55)	8 (1.04)	6 (0.78)	7 (0.86)	13 (1.68)	
Marriage status, (*n*, %)							<0.001
No spouse	2083 (51.52)	515 (56.91)	371 (48.24)	362 (46.83)	403 (49.51)	432 (55.24)	
Have a spouse	1960 (48.48)	390 (43.09)	398 (51.76)	411 (53.17)	411 (50.49)	350 (44.76)	
Self-reported quality of life, (*n*, %)							0.066
Good	2,379 (59.92)	519 (58.64)	459 (60.63)	474 (62.21)	498 (62.17)	429 (56.08)	
Bad	1,591 (40.08)	366 (41.36)	298 (39.37)	288 (37.80)	303 (37.83)	336 (43.92)	
Self-reported health, (*n*, %)							0.235
Good	1868 (47.02)	407 (45.89)	362 (47.82)	367 (48.16)	395 (49.31)	337 (44.00)	
Bad	2,105 (52.98)	480 (54.12)	395 (52.18)	395 (51.84)	406 (50.69)	429 (56.00)	
Smoking status, (*n*, %)							<0.001
No	2,584 (64.25)	623 (68.99)	479 (62.86)	480 (62.10)	480 (59.26)	522 (67.44)	
Yes	1,438 (35.75)	280 (31.01)	283 (37.14)	293 (37.90)	330 (40.74)	252 (32.56)	
Alcohol consumption, (*n*, %)							0.018
No	2,676 (66.82)	628 (70.33)	479 (62.94)	499 (65.23)	540 (66.50)	530 (68.48)	
Yes	1,329 (33.18)	265 (29.68)	282 (37.06)	266 (34.77)	272 (33.50)	244 (31.53)	
Regular exercise, (*n*, %)							0.607
No	2,158 (54.05)	475 (52.95)	408 (53.76)	423 (55.44)	449 (55.71)	403 (52.47)	
Yes	1835 (45.96)	422 (47.05)	351 (46.25)	340 (44.56)	357 (44.29)	365 (47.53)	
Hypertension, (*n*, %)							0.464
No	2,689 (68.42)	599 (67.91)	498 (66.58)	505 (67.79)	548 (69.02)	539 (70.83)	
Yes	1,241 (31.58)	283 (32.09)	250 (33.42)	240 (32.22)	246 (30.98)	222 (29.17)	
Diabetes, (*n*, %)							0.173
No	3,748 (95.86)	843 (95.36)	704 (94.62)	718 (96.12)	763 (96.22)	720 (97.04)	
Yes	162 (4.14)	41 (4.64)	40 (5.38)	29 (3.88)	30 (3.78)	22 (2.97)	
Heart disease, (*n*, %)							0.174
No	3,461 (88.40)	798 (89.97)	643 (86.66)	662 (88.62)	705 (89.35)	653 (87.07)	
Yes	454 (11.60)	89 (10.03)	99 (13.34)	85 (11.38)	84 (10.65)	97 (12.93)	
Stroke/ cerebrovascular, (*n*, %)							0.262
No	3,646 (93.03)	825 (92.91)	680 (91.89)	691 (92.26)	746 (94.55)	704 (93.49)	
Yes	273 (6.97)	63 (7.10)	60 (8.11)	58 (7.74)	43 (5.45)	49 (6.51)	
Cancer, (*n*, %)							0.514
No	3,864 (99.41)	872 (99.20)	728 (99.32)	740 (99.20)	783 (99.75)	741 (99.60)	
Yes	23 (0.59)	7 (0.80)	5 (0.682)	6 (0.80)	2 (0.26)	3 (0.40)	
Dyslipidemia, (*n*, %)							0.649
No	3,588 (96.56)	793 (96.47)	684 (95.93)	690 (96.23)	731 (96.82)	690 (97.32)	
Yes	128 (3.45)	29 (3.53)	29 (4.07)	27 (3.77)	24 (3.18)	19 (2.68)	
Survival status, *n* (%)							<0.001
Survival	2,616 (64.35)	519 (57.22)	524 (67.44)	541 (69.54)	543 (66.30)	489 (62.37)	
Death	1,449 (35.65)	388 (42.78)	253 (32.56)	237 (30.46)	276 (33.70)	295 (37.63)	

Total percentages within categories may not sum to 100% due to rounding.

### 3.2 Associations of changes in waist-to-height ratio with all-cause mortality

The study used Cox proportional hazards models to study the association between changes in WHtR and all-cause mortality. In the unadjusted model, the association between changes in WHtR and all-cause mortality was significant (HR: 0.50; 95%CI: 0.27, 0.92), and remained statistically significant after adjustment for covariates: baseline WHtR, demographic characteristics, health behaviors, and health status (HR: 0.30; 95%CI: 0.13, 0.68). In the unadjusted model, compared with participants with WHtR-stable group (change less than 0.01), participants who had a loss more than 0.06 in WHtR had 45% higher risk of all-cause mortality (HR: 1.45; 95% CI: 1.24, 1.71), and those who had a more than 0.06 increase in WHtR had a 26% higher (HR: 1.26; 95% CI: 1.06, 1.49) risk. In model 4, after adjusting for baseline WHtR, demographic characteristics, health behaviors, and health status, the value of HR decreased to 1.30 but remained statistically significant within the group of WHtR that lost more than 0.06. However, the other groups showed no statistical significance (Table 2).

**Table 2:** Associations of changes in waist-to-height ratio and the categories of changes in waist-to-height ratio with all-cause mortality among older adults.

Model	Model 1			Model 2		Model 3		Model 4	
	*N*	HR (95% CI)	*P*	HR (95% CI)	*P*	HR (95% CI)	*P*	HR (95% CI)	*P*
WHtR changes	4,065	0.50 (0.27, 0.92)	0.025	0.12 (0.06, 0.25)	<0.001	0.28 (0.13, 0.58)	<0.001	0.30 (0.13, 0.68)	0.004
Categories of the Changes in WHtR									
≤ − 0.06	907	1.45 (1.24, 1.71)	<0.001	1.66 (1.40, 1.96)	<0.001	1.36 (1.14, 1.63)	0.001	1.30 (1.07, 1.59)	0.009
−0.05 ~ −0.02	777	1.06 (0.89, 1.26)	0.502	1.10 (0.92, 1.31)	0.285	1.06 (0.88, 1.28)	0.547	1.01 (0.82, 1.25)	0.921
−0.01 ~ 0.01	778	1 [Reference]		1 [Reference]		1 [Reference]		1 [Reference]	
0.02 ~ 0.05	819	1.11 (0.93, 1.31)	0.262	1.05 (0.88, 1.25)	0.590	0.98 (0.82, 1.19)	0.861	0.99 (0.80, 1.21)	0.907
≥ 0.06	784	1.26 (1.06, 1.49)	0.009	1.09 (0.91, 1.30)	0.341	1.00 (0.83, 1.21)	0.997	0.95 (0.77, 1.18)	0.634

Model 1 was unadjusted. Model 2 was adjusted for baseline WHtR. Model 3 was adjusted baseline WHtR, age, sex, urban–rural distribution, race, living arrangement, education level, and marriage status. Model 4 was adjusted baseline WHtR, age, sex, urban–rural distribution, race, living arrangement, education level, marriage status, self-reported quality of life, self-reported health, smoking status, alcohol consumption, regular exercise, hypertension, diabetes, heart disease, stroke or cerebrovascular, cancer, and dyslipidemia.

WHtR, waist-to-height ratio. HR, hazard ratio. CI, confidence interval.

**Figure 2:**
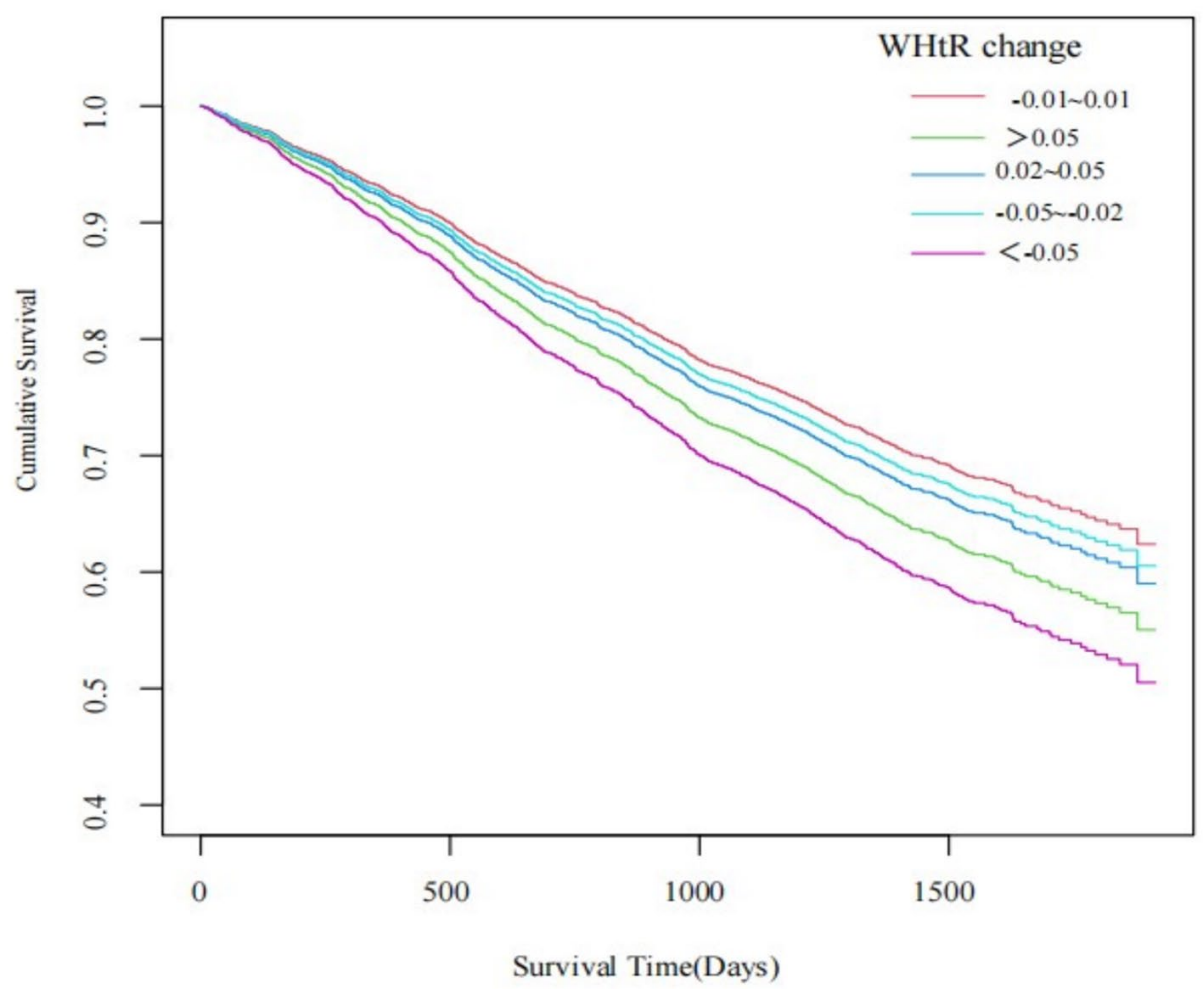
Kaplan–Meier estimates of all-cause mortality by categories of changes in waist-to-height ratio.

The Log-rank statistic showed significant differences in survival time between the categories of the changes in WHtR (*p* < 0.001), with participants having the largest decrease in WHtR showing the lowest cumulative survival (Figure 2).

### 3.3 Stratification analysis

The results of stratified analyses by sex, race, self-reported quality of life, self-reported health, alcohol consumption, smoking status, regular exercise, hypertension, diabetes, heart disease, stroke or cerebrovascular, cancer, and dyslipidemia were exhibited (see Supplementary Table 1). The interaction results showed that the association between the changes in WHtR and all-cause mortality did not differ among the study subgroups (all *P*-interactions > 0.05).

### 3.4 Sensitivity analyses

First, we tested the result using multiple imputations for missing data (see Supplementary Table 2). Second, after excluding participants whose survival time was less than 6 months (*n* = 56), the estimates and the significance levels of the observed associations were not altered in the five groups of the changes in WHtR (see Supplementary Table 3). Moreover, excluding participants who suffered from diabetes or dyslipidemia (*n* = 251), the direction of the results did not alter (see Supplementary Table 4). Those sensitivity analyses were all reasonably consistent with the main text. Finally, we investigated the associations of BMI changes with all-cause mortality (*n* = 3,945). Among these participants, when BMI changes were used as a continuous variable, there was no significant association between BMI changes and all-cause mortality. However, when BMI changes were treated as a 5-group variable, the association of BMI changes with all-cause mortality remained statistically significant in participants who lost more than 10% in BMI change (*p* < 0.05) (see Supplementary Table 5).

## 4 Discussion

The study investigated the associations between the changes in WHtR and all-cause mortality among Chinese older adults. This study revealed a significant negative associations between the changes in WHtR and all-cause mortality when the changes in WHtR were used as a continuity variable. When the changes in WHtR were used as a categorical variable, compared to participants in the WHtR-stable group (change less than 0.01), participants with a loss of more than 0.06 in WHtR were significantly associated with all-cause mortality. And the current study showed that the association between the changes in WHtR and all-cause mortality stayed stable across the study subgroups.

A previous study has reported an increased risk of mortality associated with low WHtR (30). Our study enriched and broadened previous research. In terms of the magnitude of the changes in WHtR, this study found an association between decreased WHtR (more than 0.06) and elevated all-cause mortality risk in comparison to the WHtR-stable group (change less than 0.01). After controlling for all the study covariates, the study found that there is no statistically significant association between an increase in WHtR and all-cause mortality. The previous findings of Butt et al. (31) indicated that higher WHtR was not associated with all-cause mortality. However, a study on participants over 30 years old in Tehran indicated that a high WHtR was associated with an increased risk of all-cause mortality (32). The controversy over these findings may stem from the different sample size, age, sex, and nationalities of participants. These studies did not comprehensively exclude participants with cancer, cardiovascular diseases, or other chronic debilitating conditions. The current study controlled for covariates of cancer, heart disease, stroke, diabetes, hypertension and dyslipidaemia. Additionally, the study specifically excluded participants with diabetes and dyslipidemia, as well as those whose survival time was less than 6 months (where the likelihood of death was predominantly due to pre-existing health conditions). Despite these exclusions, the observed association persisted.

Previous studies have established the association between various anthropometric obesity indicators and all-cause mortality. WHtR, akin to anthropometric measures such as weight, waist circumference, and BMI, serves as a pertinent indicator for assessing adiposity. It shares similarities with the association of changes in weight, waist circumference, and BMI with all-cause mortality. Besides, the changes in WHtR are easier to measure and calculate than other indices, and its characteristic of considering both waist circumference and height parameters makes it a more comprehensive assessment of fat and muscle distribution, and higher sensitivity in predicting a variety of health outcomes.

The association between weight loss and all-cause mortality among old adults has been recognized by most scholars. Some studies have found that weight loss has been associated with increased mortality risk (16, 33, 34). However, there was a study showed no association between weight loss and all-cause mortality in older adults (35). Meanwhile, there has been ongoing controversy about the association between weight gain and all-cause mortality in old adults. Some studies indicated an association between weight gain and heightened all-cause mortality risk (36, 37), while others found no association between weight gain and all-cause mortality in older adults (38, 39). The latter is in line with the results of the current study, where WHtR gain was not associated with all-cause mortality. These different results may be due to differences in demographic and cultural factors between studies, such as age, sex, and race (40). During the aging process, the changes in WHtR are different in different age groups and may lead to considerable absolute errors due to some factors, such as posterior convexity deformities and edema. In addition, sex-specific physiological differences and health behaviors as well as ethnogenetically related lifestyles may contribute to the variability of study results. The interaction of these factors increases the complexity of the changes in WHtR and its impact on all-cause mortality. Future studies should further consider these factors to improve the accuracy and applicability of the results of this study.

Waist circumference is a key variable in the calculation of WHtR and may be a better measure than weight for estimating all-cause mortality. Change in waist circumference as one of the important indicators may play a key role in explaining the association between changes in WHtR and all-cause mortality. The majority of studies confirmed a significant association between waist circumference decline and increased all-cause mortality (16, 41). However, the association between waist circumference increase and all-cause mortality was uncertain. Some studies suggested that an increase in waist circumference was unrelated to all-cause mortality (16, 42), while others showed an association with elevated all-cause mortality (22, 43). This difference may be explained by the fact that the higher mortality associated with increased waist circumference may stem from a gain in harmful abdominal fat. Change in waist circumference directly reflects dynamic changes in abdominal adiposity, providing a more nuanced understanding of the physiological basis that leads to changes in WHtR associated with elevated all-cause mortality.

The BMI is the most commonly used measure of obesity, and an association between changes in BMI and all-cause mortality is controversial. Previous studies on association between changes in BMI and all-cause mortality in older adults have reported different results. A study demonstrated that a decrease in BMI increased all-cause mortality (24), while others have shown that an increase in BMI was not associated with or increased all-cause mortality (33, 39). In this study, changes in BMI were associated with mortality primarily at the extremes, with elevated risks observed at both extreme weight loss and, to a lesser extent, weight gain, while the continuous analysis was null. One possible explanation was that BMI cannot distinguish between fat mass, lean mass, and fluid-related fluctuations (44, 45), which may attenuate true associations with health outcomes. In contrast, changes in WHtR were associated with increased mortality mainly in the group with the largest decrease. In addition, the continuous analysis demonstrated a statistically significant association between changes in WHtR and mortality. This may be because WHtR more directly reflects central adiposity and visceral fat accumulation (2), which are more closely related to metabolic dysfunction and adverse health outcomes in older adults, particularly in Asian populations where central obesity is more prevalent even at relatively lower BMI levels (46). Therefore, changes in WHtR may capture central fat redistribution and related metabolic risk in older adults, and substantial reductions in WHtR may be associated with increased mortality risk. However, these findings should be interpreted with caution.

When the changes in WHtR was used as a continuous variable, it was negatively associated with all-cause mortality. A study focusing on older adults over 80 years with overweight and abdominal obesity found that human measures of abdominal fat accumulation estimated by WHtR were inversely associated with mortality (13). This phenomenon might be explained by the fact that older adults often suffer from chronic diseases, and the accumulation of abdominal fat may provide energy reserves to cope with certain diseases or physical stress, which is consistent with the so-called “obesity paradox,” but the detailed mechanisms need to be further investigated. Changes in WHtR reflect altered abdominal fat distribution in older adults, which is a key factor in the assessment of all-cause mortality.

The underlying mechanisms between decreased WHtR or weight loss and high all-cause mortality among older adults are a complex result of the combined various factors. The weight loss can serve as an early predictive indicator for various life-shortening diseases (47, 48). First, a previous studies have reported an association between WHtR and sarcopenia in older adults (49), and a longitudinal evidence has further suggested that elevated baseline WHtR may predict the incidence of sarcopenic obesity (50). Additionally, sarcopenia is associated with an increased all-cause mortality (51). However, it is important to note that existing evidence was largely derived from cross-sectional analyses, and longitudinal data examining whether temporal changes in WHtR were directly associated with incident sarcopenia remain limited. With age increasing, muscle loss is common and regarded as a new geriatric syndrome (52, 53). The reduction of muscle mass as well as body energy reserves, may contribute to a decrease in basal metabolic rate and physical capability (54, 55), which is associated with poorer health of the body, thereby increasing the risk of all-cause mortality (56, 57). Second, changes in WHtR in older adults can be attributed to the progression of common chronic diseases, which often trigger a cascade of health problems in advanced stages (58). Chronic diseases progressively deplete the body of energy, and this process will be accelerated by a variety of causes (e.g., poor appetite and insufficient calorie intake). Consequently, an obvious result of this health deterioration is malnutrition, leading to lean body mass (59). Lean body mass not only interferes with normal physiological functions of the body, but also triggers change in fat distribution, contributing to the changes in WHtR, which in turn increases the risk of all-cause mortality (60). Furthermore, mental health may be an important factor. Depression and anxiety contribute to an increased metabolic rate, diminished food intake, and decreased utilization efficiency of carbohydrate storage, resulting in lean body mass and triggering changes in WHtR (61), thereby increasing the risk of all-cause mortality.

These mechanisms do not exist separately but interact with each other, future research is needed to further elucidate the potential mechanisms of association of the changes in WHtR with all-cause mortality and to provide targeted interventions to maintain optimal weight and body composition in older adults and promote healthy aging.

The present study has some limitations that should be considered. Firstly, intentional and unintentional changes in WHtR were not considered in this study because data on such variables were not available. However, this distinction is critical because intentional and unintentional changes in WHtR may have different effects on all-cause mortality. Future studies need to further explore and clarify the association between intentional and unintentional changes in WHtR and all-cause mortality in older adults. Secondly, due to the fast and easy measurement of WHtR, it is more commonly used in clinical practice. But it does not capture the precise changes in fat versus muscle. If change in body composition wants to be measured accurately, future studies need to consider incorporating advanced imaging techniques (e.g., CT or MRI) to measure change in body composition, which would allow for a more detailed exploration of the association between change in body composition and all-cause mortality. Thirdly, in this study, disease assessment was based on participants’ self-reports, and therefore classification errors may have occurred. Fourthly, as this is a longitudinal cohort study requiring complete follow-up data. This inevitably resulted in a reduction in sample size compared with the baseline cohort, which may limit the representativeness of the study population. Future studies with more complete follow-up data and larger sample sizes are warranted to further validate these findings. Fifthly, although efforts were made to recruit a broad range of participants, some degree of selection bias cannot be entirely excluded. This is partly attributable to the fact that anthropometric measurements were conducted during home visits, which may have resulted in the underrepresentation of individuals with severe illness or those who were institutionalized, potentially leading to an underestimation of the observed associations. Finally, although a series of potential confounding factors were controlled, residual and unmeasured confounders, such as undiagnosed chronic diseases and dietary changes cannot be completely excluded. Future studies should consider exploring potential confounders more comprehensively to increase the robustness of the study.

## 5 Conclusion

The study found that the changes in WHtR were inversely associated with all-cause mortality, and compared with participants of WHtR-stable group (change less than 0.01), participants who had a loss more than 0.06 in WHtR had a higher risk of all-cause mortality. The study not only provided insights into understanding the complex association between changes in anthropometric measures and all-cause mortality but also stressed the importance of maintaining WHtR in older adults. The use of the changes in WHtR will have a better application in identifying the risk of health status in older adults.

## Data Availability

The original contributions presented in the study are included in the article/Supplementary material, further inquiries can be directed to the corresponding author.

## Footnotes

1
http://www.R-project.org
