# Supplementary material for: Associations of changes in waist-to-height ratio with all-cause mortality among Chinese older adults: a national cohort study
Source: Front Nutr. 2026 May 14;13:1839686. doi: 10.3389/fnut.2026.1839686 (PMC13215858; doi:10.3389/fnut.2026.1839686)
Supplement: Supplementary file 1 [file Table_1.docx]

Supplementary Table 1. Association of the categories of changes in waist-to-height ratio with all-cause mortality, stratified by categorical variables.

| Subgroups | HR (95%CI) | | | | | P interaction |
| --- | --- | --- | --- | --- | --- | --- |
|  | ≤ -0.06 | -0.05 ~ -0.02 | -0.01 ~ 0.01 | 0.02 ~ 0.05 | ≥ 0.06 |  |
| Sex |  |  |  |  |  | 0.457 |
| Male | 1.33 (1.05, 1.69)* | 1.09 (0.86, 1.39) | 1 [Reference] | 1.09 (0.86, 1.39) | 1.12 (0.88, 1.44) |  |
| Female | 1.58 (1.25, 2.01) *** | 1.05 (0.79, 1.38) | 1 [Reference] | 1.15 (0.88, 1.51) | 1.43 (1.11, 1.84)** |  |
| Race |  |  |  |  |  | 0.807 |
| Other | 2.00 (0.95, 4.20) | 1.49 (0.65, 3.41) | 1 [Reference] | 1.38 (0.62, 3.07) | 1.35 (0.586, 3.13) |  |
| Han | 1.38 (1.15, 1.65)*** | 1.09 (0.89, 1.32) | 1 [Reference] | 1.12 (0.92, 1.36) | 1.24 (1.02, 1.50)* |  |
| Drinking |  |  |  |  |  | 0.575 |
| No | 1.56 (1.27, 1.91) *** | 1.12 (0.89, 1.41) | 1 [Reference] | 1.24 (1.00, 1.55) | 1.34 (1.08, 1.67)** |  |
| Yes | 1.24 (0.93, 1.66) | 0.95 (0.70, 1.28) | 1 [Reference] | 0.91 (0.67, 1.23) | 1.12 (0.83, 1.51) |  |
| Smoking |  |  |  |  |  | 0.356 |
| No | 1.55 (1.25, 1.93) *** | 1.14 (0.90, 1.45) | 1 [Reference] | 1.30 (1.03, 1.64)* | 1.41 (1.12, 1.77)** |  |
| Yes | 1.36 (1.05, 1.78)* | 0.98 (0.74, 1.29) | 1 [Reference] | 0.91 (0.69, 1.20) | 1.09 (0.82, 1.44) |  |
| Physical |  |  |  |  |  | 0.418 |
| No | 1.58 (1.26, 1.99) *** | 1.17 (0.91, 1.49) | 1 [Reference] | 1.30 (1.02, 1.65)* | 1.42 (1.11, 1.80)** |  |
| Yes | 1.349 (1.06, 1.72)* | 0.98 (0.75, 1.28) | 1 [Reference] | 0.91 (0.69, 1.19) | 1.14 (0.88, 1.48) |  |
| Hypertension |  |  |  |  |  | 0.347 |
| No | 1.36 (1.11, 1.67)** | 1.10 (0.88, 1.37) | 1 [Reference] | 1.10 (0.89, 1.37) | 1.21 (0.98, 1.49) |  |
| Yes | 1.63 (1.21, 2.21)** | 0.91 (0.65, 1.29) | 1 [Reference] | 1.13 (0.81, 1.58) | 1.39 (1.00, 1.93)* |  |
| Diabetes |  |  |  |  |  | 0.771 |
| No | 1.43 (1.21, 1.70)*** | 1.07 (0.89, 1.29) | 1 [Reference] | 1.12 (0.93, 1.34) | 1.26 (1.05, 1.52)* |  |
| Yes | 1.34 (0.60, 3.00) | 0.70 (0.28, 1.75) | 1 [Reference] | 1.25 (0.52, 3.02) | 1.16 (0.45, 3.02) |  |
| Blood disease |  |  |  |  |  | 0.471 |
| No | 1.38 (1.16, 1.65)*** | 1.00 (0.83, 1.21) | 1 [Reference] | 1.11 (0.93, 1.34) | 1.19 (0.99, 1.43) |  |
| Yes | 1.64 (0.48, 5.60) | 1.14 (0.31, 4.25) | 1 [Reference] | 0.80 (0.18, 3.58) | 2.65 (0.78, 9.07) |  |

Note：*P<0.05.**P<0.01. ***P<0.001.

Supplementary Table 2. Multiple imputation models on the associations of changes in waist-to-height ratio and the categories of changes in waist-to-height ratio with all-cause mortality.

| Model | Model 1 | | | Model 2 | | Model 3 | | Model 4 | |
| --- | --- | --- | --- | --- | --- | --- | --- | --- | --- |
|  | N | HR (95% CI) | P | HR (95% CI) | P | HR (95% CI) | P | HR (95% CI) | P |
| WHtR Changes | 4065 | 0.50 (0.27,0.92) | 0.025 | 0.12 (0.06,0.25) | <0.001 | 0.24 (0.12,0.49) | <0.001 | 0.24 (0.12,0.49) | <0.001 |
| Categories of Changes in WHtR | | | | | | | | | |
| ≤ -0.06 | 907 | 1.45 (1.24,1.71) | <0.001 | 1.66 (1.40,1.96) | <0.001 | 1.37 (1.16,1.62) | <0.001 | 1.36 (1.15,1.61) | <0.001 |
| -0.05 ~ -0.02 | 777 | 1.06 (0.89,1.27) | 0.502 | 1.10 (0.92,1.32) | 0.285 | 1.04 (0.87,1.24) | 0.697 | 1.03 (0.86,1.23) | 0.770 |
| -0.01 ~ 0.01 | 778 | 1 [Reference] |  | 1 [Reference] |  | 1 [Reference] |  | 1 [Reference] |  |
| 0.02 ~ 0.05 | 819 | 1.11 (0.93,1.31) | 0.262 | 1.05 (0.88,1.25) | 0.590 | 0.97 (0.82,1.16) | 0.761 | 0.97 (0.81,1.16) | 0.738 |
| ≥ 0.06 | 784 | 1.26 (1.06,1.49) | 0.009 | 1.09 (0.91,1.30) | 0.341 | 0.99 (0.83,1.18) | 0.877 | 0.98 (0.82,1.17) | 0.784 |

Note: Model 1 was unadjusted. Model 2 was adjusted for baseline WHtR. Model 3 was adjusted baseline WHtR, age, sex, urban-rural distribution, race, living arrangement, education level, and marriage status. Model 4 was adjusted baseline WHtR, age, sex, urban-rural distribution, race, living arrangement, education level, marriage status, self-reported quality of life, self-reported health, smoking status, alcohol consumption, regular exercise, hypertension, diabetes, heart disease, stroke or cerebrovascular, cancer, and dyslipidemia.

Abbreviations: WHtR, waist-to-height ratio. HR, hazard ratio. CI, confidence interval.

Supplementary Table 3. Associations of changes in waist-to-height ratio and the categories of changes in waist-to-height ratio with all-cause mortality excluding participants whose survival time was less than 6 months (n＝4011).

| Model | Model 1 | | | Model 2 | | Model 3 | | Model 4 | |
| --- | --- | --- | --- | --- | --- | --- | --- | --- | --- |
|  | N | HR (95% CI) | P | HR (95% CI) | P | HR (95% CI) | P | HR (95% CI) | P |
| WHtR Changes | 4011 | 0.58 (0.31,1.08) | 0.084 | 0.15 (0.07,0.31) | <0.001 | 0.32 (0.15,0.68) | 0.003 | 0.36 (0.15,0.85) | 0.020 |
| Categories of Changes in WHtR | | | | | | | | | |
| ≤ -0.06 | 882 | 1.37 (1.16,1.62) | <0.001 | 1.56 (1.31,1.85) | <0.001 | 1.28 (1.06,1.53) | 0.009 | 1.24 (1.01,1.51) | 0.040 |
| -0.05 ~ -0.02 | 769 | 1.04 (0.87,1.24) | 0.709 | 1.07 (0.90,1.29) | 0.436 | 1.02 (0.84,1.24) | 0.816 | 0.97 (0.79,1.21) | 0.812 |
| -0.01 ~ 0.01 | 776 | 1 [Reference] |  | 1 [Reference] |  | 1 [Reference] |  | 1 [Reference] |  |
| 0.02 ~ 0.05 | 810 | 1.08 (0.90,1.28) | 0.404 | 1.03 (0.86,1.22) | 0.783 | 0.96 (0.80,1.16) | 0.687 | 0.97 (0.79,1.19) | 0.757 |
| ≥ 0.06 | 774 | 1.22 (1.03,1.46) | 0.022 | 1.07 (0.89,1.28) | 0.471 | 0.98 (0.81,1.19) | 0.873 | 0.95 (0.76,1.18) | 0.628 |

Note: Model 1 was unadjusted. Model 2 was adjusted for baseline WHtR. Model 3 was adjusted baseline WHtR, age, sex, urban-rural distribution, race, living arrangement, education level, and marriage status. Model 4 was adjusted baseline WHtR, age, sex, urban-rural distribution, race, living arrangement, education level, marriage status, self-reported quality of life, self-reported health, smoking status, alcohol consumption, regular exercise, hypertension, diabetes, heart disease, stroke or cerebrovascular, cancer, and dyslipidemia.

Abbreviations: WHtR, waist-to-height ratio. HR, hazard ratio. CI, confidence interval.

Supplementary Table 4. Associations of changes in waist-to-height ratio and the categories of changes in waist-to-height ratio with all-cause mortality excluding participants who suffered from diabetes or dyslipidemia (n＝3814).

| Model | Model 1 | | | Model 2 | | Model 3 | | Model 4 | |
| --- | --- | --- | --- | --- | --- | --- | --- | --- | --- |
|  | N | HR (95% CI) | P | HR (95% CI) | P | HR (95% CI) | P | HR (95% CI) | P |
| WHtR Changes | 3814 | 0.44 (0.24,0.81) | 0.009 | 0.12 (0.06,0.24) | <0.001 | 0.24 (0.11,0.51) | <0.001 | 0.23 (0.10,0.53) | 0.001 |
| Categories of Changes in WHtR | | | | | | | | | |
| ≤ -0.06 | 846 | 1.45 (1.23,1.71) | <0.001 | 1.64 (1.38,1.95) | <0.001 | 1.37 (1.14,1.64) | <0.001 | 1.33 (1.09,1.61) | 0.005 |
| -0.05 ~ -0.02 | 717 | 1.07 (0.89,1.28) | 0.476 | 1.10 (0.92,1.33) | 0.26 | 1.08 (0.89,1.32) | 0.436 | 1.04 (0.84,1.28) | 0.743 |
| -0.01 ~ 0.01 | 730 | 1 [Reference] |  | 1 [Reference] |  | 1 [Reference] |  | 1 [Reference] |  |
| 0.02 ~ 0.05 | 773 | 1.09 (0.91,1.30) | 0.345 | 1.05 (0.87,1.24) | 0.668 | 0.98 (0.81,1.19) | 0.838 | 0.97 (0.79,1.19) | 0.745 |
| ≥ 0.06 | 748 | 1.23 (1.03,1.47) | 0.020 | 1.08 (0.90,1.30) | 0.401 | 0.98 (0.80,1.19) | 0.819 | 0.94 (0.76,1.16) | 0.562 |

Note: Model 1 was unadjusted. Model 2 was adjusted for baseline WHtR. Model 3 was adjusted baseline WHtR, age, sex, urban-rural distribution, race, living arrangement, education level, and marriage status. Model 4 was adjusted baseline WHtR, age, sex, urban-rural distribution, race, living arrangement, education level, marriage status, self-reported quality of life, self-reported health, smoking status, alcohol consumption, regular exercise, hypertension, diabetes, heart disease, stroke or cerebrovascular, cancer, and dyslipidemia.

Abbreviations: WHtR, waist-to-height ratio. HR, hazard ratio. CI, confidence interval.

Supplementary Table 5. Associations of changes in body mass index and the categories of changes in body mass index with all-cause mortality (n＝3945).

| Model | Model 1 | | | Model 2 | | Model 3 | | Model 4 | |
| --- | --- | --- | --- | --- | --- | --- | --- | --- | --- |
|  | N | HR (95% CI) | P | HR (95% CI) | P | HR (95% CI) | P | HR (95% CI) | P |
| BMI Changes | 3945 | 1.00 (0.99,1.01) | 0.717 | 1.00 (0.98,1.01) | 0.625 | 1.00 (0.98,1.01) | 0.677 | 1.00 (0.98,1.02) | 0.946 |
| Categories of BMI Changes | | | | | | | | | |
| < -10% | 700 | 1.58 (1.35,1.84) | <0.001 | 1.58 (1.35,1.85) | <0.001 | 1.35 (1.14,1.60) | <0.001 | 1.33 (1.10,1.62) | 0.003 |
| -10% ~ -5% | 568 | 1.20 (1.01,1.43) | 0.042 | 1.20 (1.01,1.44) | 0.040 | 1.14 (0.95,1.38) | 0.169 | 1.16 (0.94,1.43) | 0.160 |
| -5% ~ 5% | 1139 | 1 [Reference] |  | 1 [Reference] |  | 1 [Reference] |  | 1 [Reference] |  |
| 5% ~ 10% | 588 | 1.17 (0.98,1.40) | 0.081 | 1.17 (0.98,1.39) | 0.084 | 1.06 (0.87,1.28) | 0.556 | 1.05 (0.85,1.30) | 0.637 |
| > 10% | 950 | 1.41 (1.22,1.64) | <0.001 | 1.41 (1.21,1.63) | <0.001 | 1.16 (0.99,1.36) | 0.060 | 1.18 (0.99,1.41) | 0.063 |

Note: Model 1 was unadjusted. Model 2 was adjusted for baseline BMI. Model 3 was adjusted baseline BMI, age, sex, urban-rural distribution, race, living arrangement, education level, and marriage status. Model 4 was adjusted baseline BMI, age, sex, urban-rural distribution, race, living arrangement, education level, marriage status, self-reported quality of life, self-reported health, smoking status, alcohol consumption, regular exercise, hypertension, diabetes, heart disease, stroke or cerebrovascular, cancer, and dyslipidemia.

Abbreviations: BMI, body mass index. HR, hazard ratio. CI, confidence interval.
